# Randomized clinical trial comparing intravitreal aflibercept combined with subthreshold laser to intravitreal aflibercept monotherapy for diabetic macular edema

**DOI:** 10.1038/s41598-022-14444-y

**Published:** 2022-06-23

**Authors:** Tomoaki Tatsumi, Yoko Takatsuna, Toshiyuki Oshitari, Tomomi Kaiho, Yohei Kawasaki, Yuki Shiko, Takeshi Sugawara, Takayuki Baba, Shuichi Yamamoto

**Affiliations:** 1 Department of Ophthalmology and Visual Science, Chiba University Graduate School of Medicine, 1-8-1, Inohana, Chuo-ku, Chiba, Chiba 260-8670 Japan; 2 Department of Ophthalmology, Chiba Rosai Hospital, 2-16, Tatsumidaihigashi, Ichihara, Chiba 290-0003 Japan; 3 Department of Ophthalmology, International University of Health and Welfare School of Medicine, 4-3, Kozunomori, Narita, Chiba 286-8686 Japan; 4 Biostatistics Section, Clinical Research Center, Chiba University Hospital, 1-8-1 Inohana, Chuo-ku, Chiba, 260-8670 Japan; 5 Faculty of Nursing, Japanese Red Cross College of Nursing, 4-1-3, Hiroo, Shibuya-ku, Tokyo, 150-0012 Japan; 6 Chiba University Hospital Translational Research and Development Center, 1-8-1, Inohana, Chuo-ku, Chiba, Chiba 260-8670 Japan

**Keywords:** Diseases, Medical research

## Abstract

To compare the efficacy and safety of intravitreal aflibercept with three loading doses + pro re nata regimen combined with subthreshold laser application to that of IVA monotherapy on eyes with diabetic macular edema. This was a phase 4 clinical trial with a prospective, randomized, and parallel investigator-driven protocol. Patients with DME were randomly assigned to the IVA monotherapy group (n = 25) or the IVA + SL combination therapy group (n = 26). The main outcome measures were the number of IVA injections and the changes in the best-corrected visual acuity (BCVA) and the central retinal thickness (CRT) at the final evaluation at 96 weeks. The mean number of IVA injections in the monotherapy group was 5.86 ± 2.43 and it was 6.05 ± 2.73 in the IVA + SL group at 96 weeks, and this difference was not significant *(P* = 0.83). The differences in the mean changes of the CRT (*P* = 0.17) and the BCVA (*P* = 0.31) were also not significant between the two groups throughout the follow-up period. We conclude that adjunct of SL to anti-VEGF therapy does not reduce the number of necessary intravitreal injections.

## Introduction

Diabetic macular edema (DME) is a common cause of a reduction of vision in eyes with non-proliferative diabetic retinopathy^1^. Yau et al*.* performed a meta-analysis of individual-level data from 35 population-based worldwide studies and reported that 6.81% of 22,896 patients with diabetes had DME^2^. The global standard therapy for center-involved DME is intravitreal injections of anti-vascular endothelial growth factor (anti-VEGF) agents^3–6^. However, Bresller et al. showed in a post hoc analyses of a clinical trial, the Diabetic Retinopathy Clinical Research Network Protocol T, that the DME can persist even after the anti-VEGF treatment in approximately 31.6 to 65.6% of the diabetic patients^7^. In addition, the results of a 2-year randomized clinical trial of anti-VEGF treatments for 660 DME patients by Wells et al. showed that 84% of the eyes had received at least 1 injection in the second year, and 98% of the protocol-required injections, based on visual acuity and OCT, were given over the 2 year study period. Even after the DME was resolved, there was a high rate of recurrences^8^. Thus, frequent anti-VEGF injections were required to maintain the resolution of the macular edema (ME).

Before anti-VEGF treatments were approved, focal and grid laser photocoagulations were the major treatment for DME^9^. However, these laser treatments were performed with a continuous wave laser that caused damage to the neural retina by the spread of thermal energy from the retinal pigment epithelium (RPE). In addition, conventional laser photocoagulation caused visible laser scars that can continue to enlarge the following photocoagulation, and the photoreceptors adjacent to the targeted laser spot were damaged and lost. In addition, choroidal neovascularizations and subretinal fibrosis have been reported to develop after continuous wave laser treatments^10–13^. However, with shorter exposure times, e.g., < 1 ms, the RPE is still affected but with less damage or no damage on the neural retina and choriocapillaris^14^.

Non-damaging laser treatments, also called subthreshold laser (SL) treatments, are currently being used to treat DME but they still elicit cellular stress responses.

There are two methods to apply the SL treatments. First, a short pulse of a continuous wave laser with a pattern-scan laser (PASCAL) and an endpoint management (EPM) algorithm^15^; and second, a subthreshold micropulse laser that deliver pulses whose duration is in the microseconds^14,16^. The use of these laser treatments limits the spread of the heat to adjacent retinal layers, and It has been reported that both types of treatments^17–19^ are effective in resolving the DME^20–27^.

A subthreshold micropulse laser oscillates the laser in a pulsed manner in units of 100 microseconds. When the exposure time becomes extremely short, the temperature rise is localized to the RPE and does not spread to the adjacent tissues^14^. The mechanism of action of the micropulse laser is believed to be the induction of an intracellular biological factor that stimulates the RPE, activates the pumping function of the RPE, and improves the macular edema^28^. Inagaki et al. confirmed the expression of heat shock protein (Hsp) by cultured pigment epithelial cells after irradiation with a micropulse laser. The expression of Hsp is known to increase with thermal stimulation, and it is a protein that protects cells^29^. They suggested that the mechanism of action of subthreshold micropulse lasers is its activation of the pigment epithelial cells by Hsp which then leads to the reduction of the DME.

Our department has been interested in determining the best treatment of DME, and in an earlier retrospective case series study, we found that an intravitreal injection of an anti-VEGF agent combined with subthreshold micropulse laser photocoagulation was effective in reducing the degree of DME^30^. In addition, Gawęcki reviewed nine studies evaluating the results of SL therapy for DME and RVO and reported that SL plus anti-VEGF might require fewer intravitreal injections than anti-VEGF monotherapy with equally good functional and morphological results. However, he concluded that the number of cases was too few to provide a reliable assessment of the effects of the combined therapy and its relation to intravitreal monotherapy, and whether the SL combined with anti-VEGF agents had a synergistic effect in treating DME was not determined^31^.

Thus, the purpose of this clinical trial was to determine the efficacy and safety of anti-VEGF therapy combined with SL treatment compared to anti-VEGF monotherapy in treating DME.

## Results

### Patients

**Figure 1:**
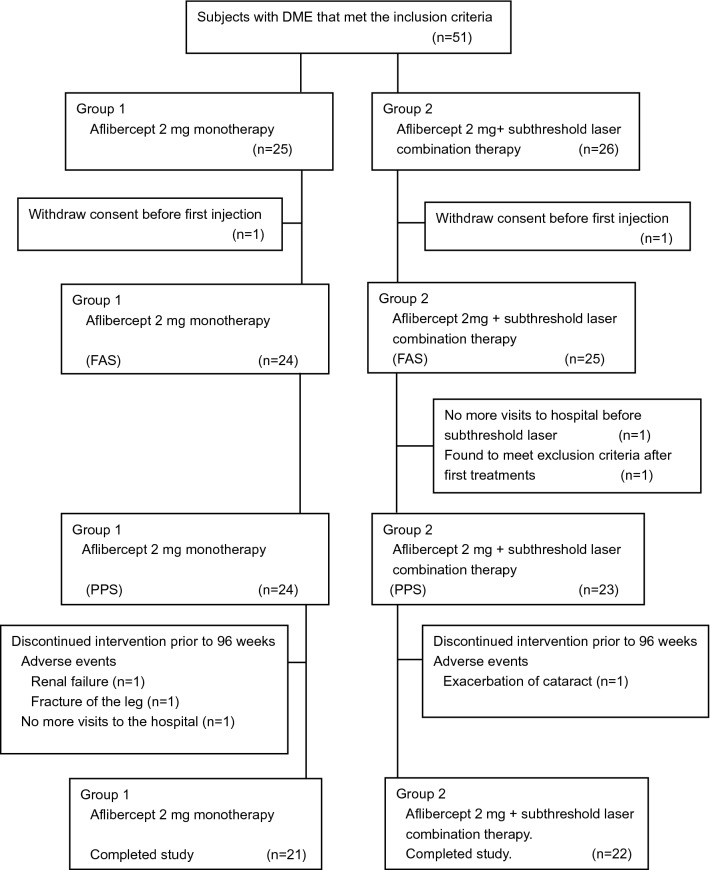
Flow diagram for selection of patients. *DME* diabetic macular edema; *FAS* full analysis set; *PPS* per protocol set.

A flow diagram for the selection of the patients is presented in Fig. 1.

Fifty-one patients were randomly assigned to two treatment groups: 25 in the intravitreal aflibercept (IVA) monotherapy group and 26 in the IVA + SL combination therapy group. One subject in each group withdrew their consent before the start of treatments, and the full analysis set (FAS) was 24 patients in the monotherapy group and 25 patients in the combination therapy group. The per protocol set (PPS) was 24 patients in the monotherapy group and 23 patients in the combination therapy group. Among the combined therapy group, subthreshold micropulse laser (577 nm micropulse laser delivered with IQ 577; IRIDEX Corporation, Mountain View, CA, USA) was performed on 6 patients and PASCAL with EPM (a continuous wave 577 nm laser with short pulses delivered with PASCAL streamline yellow, Topcon, Tokyo, Japan) was performed on 17 patients. In the PPS, 3 patients in the monotherapy group visited the hospital at 40, 48, and 76 weeks, and 1 patient in the combination therapy group visited hospital for 36 weeks after which they discontinued to visit hospital. In the end, 21 patients in the IVA monotherapy group and 22 in the IVA + SL combination therapy group completed the study of 96 weeks. In the combined therapy group, subthreshold micropulse laser was performed in 6 patients and PASCAL with EPM was performed in 16 patients.

The baseline characteristics of patients are presented in Supplementary Table S1. The differences in the mean intervals between the DME diagnosis and the first IVA injection between the two groups was not significant. However, there was a significant difference in the distribution of the mean intervals between the DME diagnosis and the first IVA. There were no significant differences in all other characteristics.

### Primary outcome

**Figure 2:**
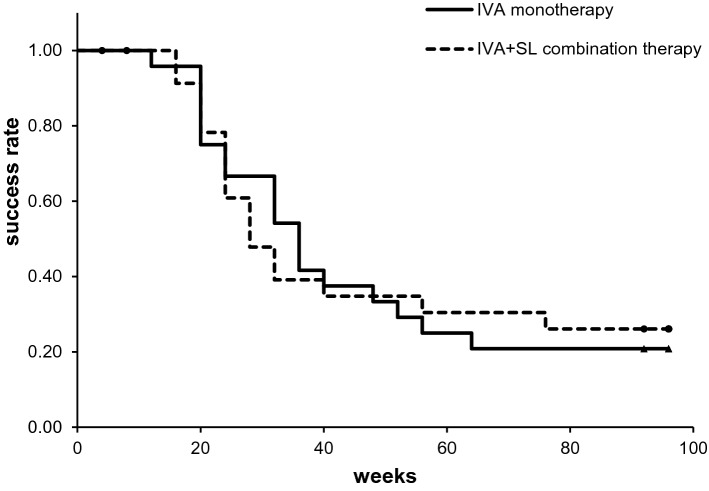
Kaplan–Meier survival curve analysis of IVA monotherapy group (n = 24) and IVA + SL combination group (n = 25) which classified retreatment due to recurrence of ME as a failure. *IVA* intravitreal injection of aflibercept; *SL* subthreshold laser.

The primary outcome was the time of the first retreatment because of a recurrence of the DME after the loading phase of 3 consecutive monthly injections of IVA with or without SL treatment. For this evaluation, Kaplan–Meier survival curves for the two treatment protocols were constructed (Fig. 2). Our results showed that the mean time of the first retreatment in the monotherapy group was 46.0 weeks, and it was 47.1 weeks in the combination group (*P* > 0.05). The success rate at 48 weeks was 37.5% (9/24 patients) in the monotherapy group and 36.0% (9/25 patients) in the combination therapy group. The success rate at 96 weeks was 20.8% (5/24 patients) in the monotherapy group and 24.0% (6/25 patients) in the combination therapy group. The differences in the success rates between the two groups were not significant (*P* = 0.98; log-rank test).

### Secondary outcomes for efficacy

#### Number of IVA injections

The mean number of IVA injections for the study period was compared between the two groups. The mean ± standard deviation (SD) number of IVA injections after 48 weeks in the IVA monotherapy group (n = 23) was 4.30 ± 1.36 and it was 4.48 ± 1.34 in the IVA + SL combined therapy group (n = 23). There was no significant difference in the number of injections (rate ratio (RR) = 0.97; 95% CI: 0.74–1.28, *P* = 0.83; Poisson regression) between the two groups.

The number of IVA for the 96 weeks study period was 5.86 ± 2.43 for the IVA monotherapy group (n = 21), and it was 6.05 ± 2.73 for the IVA + SL combination therapy group (n = 22). The difference in the number of injections was not significant (RR = 0.98; 95% CI: 0.77–1.24, *P* = 0.857; Poisson regression). The PPS was used as data in this analysis, and it excluded patients who discontinued the treatments before the 48- and 96-week study period. The number of IVA injections in both groups from the start to the 48 and 96 weeks is shown in Supplementary Figure S1a and S1b.

#### Central retinal thickness (CRT)

The CRTs at the baseline was 442.8 ± 91.3 µm, and it was 347.3 ± 58.9 µm at 48 weeks and 319.5 ± 52.2 μm at 96 weeks in the IVA monotherapy group. There was a significant decrease from baseline at both 48 and 96 weeks (both *P* < 0.0001, paired *t* tests). In the IVA + SL combination group, the CRT at the baseline was 472.8 ± 136.1 µm, it was 344.7 ± 73.1 µm at 48 weeks, and 329.0 ± 78.5 μm at 96 weeks. There was a significant decrease from baseline at both 48 (0.0006) and 96 weeks (*P* = 0.0002, paired *t* tests).

**Figure 3:**
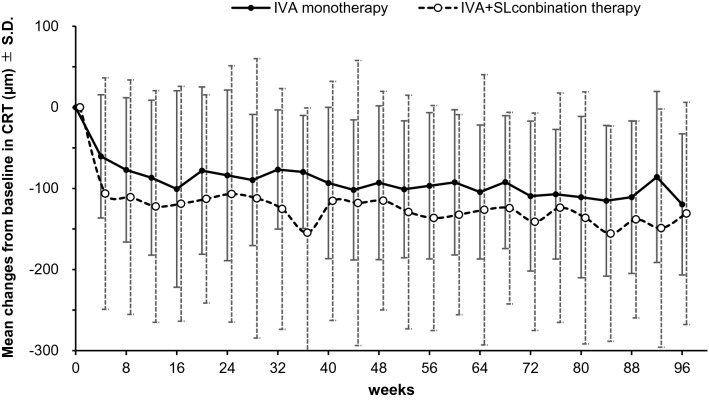
Mean changes of CRT from baseline in the IVA monotherapy group and IVA + SL combination therapy group. *IVA* intravitreal aflibercept; *SL* subthreshold laser; *CRT* central retinal thickness. *SD* standard deviation.

The mean changes of the CRT from the baseline to that at 48 weeks and at 96 weeks are plotted in Fig. 3. The mean change of the CRT in the IVA group (n = 23) was − 93.0 ± 94.9 µm and it was − 115.0 ± 134.7 μm in the IVA + SL group (n = 23) after 48 weeks. At 96 weeks, the mean change of the CRT in the IVA group (n = 21) was − 120.0 ± 87.0 and it was − 131.0 ± 137.0 μm in the IVA + SL group (n = 22). The differences in the changes in the CRTs were not significant after 48 weeks (diff (95%CI) = 38.2(− 33.9–10.2), *P* = 0.30) and also after 96 weeks (diff(95%CI) = 30.3(− 42.2–102.8), *P* = 0.41). In addition, there was no statistical difference throughout the follow-up period (*P* = 0.17 for treatment effects, mixed model regression analysis). In this analysis, the dataset used was the PPS and the last observation carried forward (LOCF) approach was used, and patients who were discontinued included only the values before the discontinuation.

#### Best-corrected visual acuity (BCVA)

The BCVAs at the baseline was 0.369 ± 0.235 the logarithm of the minimum angle of resolution (logMAR) units, and it was 0.219 ± 0.173 logMAR units at 48 weeks, and 0.317 ± 0.261 logMAR units at 96 weeks in the IVA monotherapy group. There was a significant decrease from baseline at both 48 (*P* < 0.0001) and 96 weeks (*P* = 0.31, paired *t* tests). In the IVA + SL group, the BCVA at the baseline was 0.478 ± 0.320 logMAR units, and it was 0.279 ± 0.222 logMAR units at 48 weeks and 0.283 ± 0.273 logMAR units at 96 weeks. There was a significant decrease from baseline at both 48 (*P* = 0.0023) and 96 weeks (*P* = 0.0087, paired *t* tests).

**Figure 4:**
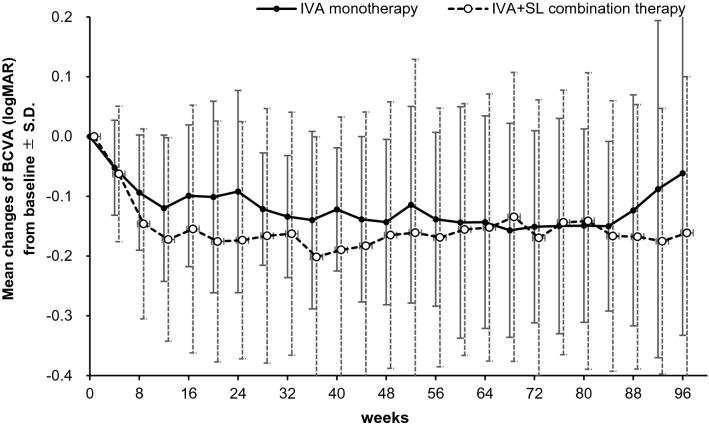
Mean changes of the BCVA from baseline in logMAR units for IVA monotherapy group and IVA + SL combination therapy group. *IVA* intravitreal aflibercept; *SL* subthreshold laser; *BCVA* best-corrected visual acuity; *logMAR* logarithm of minimum angle of resolution; *S.D.* standard deviation.

The mean changes of the BCVA from the baseline are plotted in Fig. 4. The mean change of the BCVAs of the IVA group (n = 23) was − 0.143 ± 0.138 logMAR units and it was − 0.151 ± 0.215 logMAR units in the IVA + SL group (n = 23) after 48 weeks. After 96 weeks, the change in the BCVA was − 0.061 ± 0.271 logMAR units in the IVA monotherapy group (n = 21) and − 0.161 ± 0.261 logMAR units in the IVA + SL group (n = 22) after 96 weeks. The differences in the changes of BCVA after 48 weeks (diff(95%CI) = 0.028(− 0.081–0.136), *P* = 0.61) were not significant. There was significant difference only after 96 weeks (diff(95%CI) = 0.117(0.008–0.227), *P* = 0.036), but no significant differences throughout the follow-up period (*P* = 0.31 for treatment effect; mixed model regression analysis). A decrease in the BCVAs in the monotherapy group was observed after 88 weeks (Fig. 4). This was because there were two cases in which there was a rapid and marked progression of a cataract. In this analysis, the PPS and the LOCF approach was used, and the values for the patients who discontinued were those before the discontinuation.

#### Number of subthreshold laser (SL) applications

SL was performed with the IVA injections at the time of a recurrence of the ME in the IVA + SL combination therapy group (n = 23, PPS). The protocol for this study allowed SL to be performed up to 2 times during the study period. In addition, if a recurrence of the macular edema occurred within 16 weeks after a previous recurrence, SL was not performed and only IVA was performed. SL was performed two times in 10 patients. The mean ± SD number of SL was 1.43 ± 0.51.

### Secondary outcomes for safety

There were no obvious differences in the number of adverse events between the two groups (Table 1). The three cataracts that developed were not the result of direct needle contact. Cataract surgeries were performed on 2 patients in the monotherapy group after the end of the study period, and in 1 patient in the combination therapy group during the study period. Macular holes were observed in one patient at the same time as the reduction of ME due to the IVA injection but it closed spontaneously.

**Table 1:** Adverse events (FAS).

Preferred term	IVA monotherapy group (n = 24)	IVA + SL combination therapy group (n = 25)
**Ocular local adverse events, n (%)**	4 (16.7)	2 (8.0)
Cataract	2 (8.3)	1 (4.0)
Dry eye	1 (4.2)	0
Normal tension glaucoma	1 (4.2)	0
Macular hole	0	1 (4.0)
**Non-ocular adverse events, n (%)**	4 (16.7)	3 (12.0)
Fracture	1 (4.2)	1 (4.0)
Pneumonia	1 (4.2)	0
Colon cancer	1 (4.2)	0
Renal failure	1 (4.2)	0
Myocardial infarction	0	1 (4.0)
Sudden deafness	0	1 (4.0)

*FAS* full analysis set; *IVA* intravitreal injection of aflibercept; *SL* subthreshold laser.

The systolic and diastolic blood pressure, and the pulse rate did not change significantly during the treatment and observation period in both groups. In addition, there were no cases in which the measured values increased or decreased significantly after the IVA injections. The mean changes of the intraocular pressure were not significantly different between the two groups, and no cases required treatment due to an elevation of the intraocular pressure (IOP). There were no cases of infectious diseases such as endophthalmitis during the follow-up period.

### Results of additional analyses

The number of injections was compared between the monotherapy group and the combination group only for treatment naive cases. The number of injections in the treatment naïve monotherapy group (n = 14) and the treatment naïve combination group (n = 9) were 4.25 and 4.33 at 48 weeks, 5.79 and 5.44 at 96 weeks. There was no significant difference in the number of injections between the two groups at 48 weeks (*P* = 0.88, Student’s *t* test) and 96 weeks (*P* = 0.74, Student’s *t* test) even in the treatment naïve cases.

## Discussion

This was a randomized clinical trial designed to compare the efficacy and safety of IVA monotherapy to combined IVA with SL to treat DME. Anti-VEGF treatment is the standard treatment for DME, but various problems have arisen with this therapy: recurrences of the ME, need for multiple injections, possibility of intraocular infections, and the burden of the drug costs in Japan. Therefore, it is important to reduce the number of injections in a cost-effective way.

In large-scale clinical trials of IVA treatments for DME, IVA injections were given monthly up to 48 weeks, or every 2 months after an initial 5 consecutive monthly injections^5^. In these trials, 8.4 to 12.2 IVA injections were performed during the 48-week study period. However, in the real-world clinical practice in Japan, monthly injections or fixed injections every two months is rarely selected, and the pro re nata (PRN) regimen, i.e., re-treatment at the time of a recurrence, is most often selected^32^. Furthermore, the number of initial monthly consecutive loading doses is recommended to be 5 injections based on the results of The DA VINCI study, a Phase 2 Clinical Trial^33^, and the Phase 3 VIST and VIVID studies^5^ of IVA for DME. However, in Japan, it is 1 or 3 injections^32^. On the other hand, when comparing the results of one loading dose + pro re nata (1 + PRN) regimen to three loading doses + pro re nata (3 + PRN) regimen, it was reported that the cases of 3 + PRN had better outcomes^34^. Therefore, the 3 + PRN regimen was used in our study to compare the effectiveness of IVA monotherapy and the IVA + SL combination therapy with a protocol that was closer to the real-world clinical practice.

As stated, the main purpose of this study was to determine a treatment protocol that was more effective than anti-VEGF monotherapy. We selected to test SL which affects the DME by a mechanism different from that of anti-VEGF treatment. It has been shown that the combined use of photocoagulation and anti-VEGF has no advantage over multiple anti-VEGF treatments in large-scale clinical trials^3,4^. However, even though there was no significant difference been the monotherapy and the combined therapy, the CRT tended to be reduced more with the combined than with the monotherapy. Thus, there is a possibility that the number of injections may differ depending on the protocol and retreatment criteria that are closer to the real-world clinical practice. SL was as effective as the previously treated cases as reported by the Early Treatment Diabetic Retinopathy Study (mETDRS) laser photocoagulation, and it did not cause any visible damage to the RPE^24,25^. When SL was used in combination with anti-VEGF therapy, it may have a synergistic effect with the conventional laser treatments.

The treatment regimen of the RESTORE^3^ and REVEAL^4^ studies was three anti-VEGF + PRN, and the monthly injections were continued if stable vision was not attained after 3 consecutive visits. Retreatment was performed when there was a decrease in the BCVA because of a progression of the DME as determined by the investigator but no specific criteria were given. Therefore, our retreatment criteria were based on the criteria in the extended study of VISTA and VIVID after 100 weeks from the start: (a) an increase of > 50 μm in the central subfield thickness compared with the lowest previous measurement; (b) new or persistent cystic retinal changes or subretinal fluid or persistent diffuse edema in the central subfield in the optical coherence tomography (OCT) images; (c) a loss of 5 letters in the BCVA from the best previous measurement in conjunction with any increase in the central subfield thickness or a decrease of 5 letters in the BCVA chart from that in the most recent examination^35^.

In the retreatment criteria in this study, two of the criteria in the extended study of VISTA and VIVID were changed: an increase of > 100 μm in the central subfield thickness compared with the lowest previous measurement, and a loss of 0.2 logMAR units in the BCVA from the best previous measurement in conjunction with any increase in central subfield thickness.

The retreatment criteria have become more difficult to meet but they are close to the criteria in the real-world clinical practice in Japan. These criteria were selected with the aim of providing useful information for the clinicians.

The number of anti-VEGF injections/year for the IVA monotherapy group was 4.30 ± 1.36 and it was 4.48 ± 1.34 for the IVA + SL combination therapy group. Both are slightly higher than the 3.8 ± 2.7 injections in the real-world clinical practice reported in Japan (2015–2017 in Chiba University Hospital)^36^, but less than the 7.0 ± 3.07 in the large-scale REVEAL study clinical trial for ranibizumab (Ranibizumab + laser)^4^.

The number of anti-VEGF injections in 2 years in the IVA monotherapy group was 5.86 ± 2.43 and it was 6.05 ± 2.73 in the IVA + SL combination therapy group. Both values are slightly higher than the 5.5 ± 3.6 in the real-world practice reported by the Japan Clinical Retina Study (J- CREST) group (number of anti-VEGF injections in the 2015–2017 STREAT-DMO study)^37^. The number of injections in our study was closer to that of the real-world practice.

SL is less effective in improving the DME quickly and significantly. Anti-VEGF treatments are significantly superior to the SL monotherapy in terms of therapeutic effects on the DME^38^. However, SL is believed to exerts its effects slowly^26^, and when used with the anti-VEGF treatments, it was difficult to identify the effects of SL in the clinical course of the DME.

Elman et al. examined the effects of intravitreal ranibizumab with prompt versus deferred (for ≥ 24 weeks) focal/grid laser treatment for DME. They reported that the mean changes of the BCVA was better in the deferred group than the prompt laser group^39^. These findings suggested that that focal/grid laser, which is performed in eyes with less severe macular edema, is more effective. We have obtained good results for DME by performing SL after several anti-VEGF treatments^30^. This is the basis for selecting the time for performing SL after the initial three anti-VEGF injections in the protocol of this study.

The mean changes in the CRT and BCVA from the baseline are shown in Figs. 3 and 4 for both groups. Both the CRT and the BCVA were improved in the early stages of treatment (after 8 weeks) in the combination therapy group, but there was no significant difference between the two groups. The reason why the combination group seemed to improve more is probably because the baseline value of this group was slightly poorer than that of the IVA monotherapy group. In the combination therapy group, the SL was performed 8 to 12 weeks after the initiation of this study, and it was not likely that the effects of SL appeared at this time.

There have been six reports comparing anti-VEGF monotherapy to combination therapy of anti-VEGF agents and SL for DME^40–45^. The methods and results of these studies are summarized in Table 2. Three of them were prospective studies. In all of these studies, the anti-VEGF monotherapy and combination therapy of anti-VEGF + SL were compared, and the changes of BCVA and CRT was not significantly different between the two groups. However, the number of anti-VEGF injections was significantly lower in the combination therapy group.

**Table 2:** Previous reports and this study comparing combination therapy of anti-VEGF and subthreshold laser/anti-VEGF monotherapy for DME.

Author/Journal/year	Material and Protocol Study period	Retreatment criteria	Study design Follow up periods	Patients Inclusion criteria of CRT and BCVA	CRT (µm) Baseline ⇒ Final * Difference	BCVA(logMAR) Baseline ⇒ Final * Difference	No of injections
Moisseiev E et al. 40	IVR + SML versus IVR Jan 2013–Jun 2015		Retrospective 12 months	n = 38 (19 vs matched control 19) All patients had no more than 3 prior IVR	IVR 408.4 ⇒ 335.9 72.5 IVR + SML 316.8 ⇒ 282.6 34.2	0.41 ⇒ 0.39 0.02 0.29 ⇒ 0.24 0.05	5.6 / 12 months 1.7 / 12 months
Altınel MG, et al. 45	IVB + SML versus IVB Sep 2017—Mar 2020		Retrospective 15 months	n = 80 (40 vs. 40) Excluded Intravitreal injections within the preceding 6 months CRT > 250 µm,	IVB 384.68 ⇒ 325.8 58.88 IVB + SML 379.2 ⇒ 292.64 86.56	0.39 ⇒ 0.32 0.07 0.38 ⇒ 0.25 0.13	8.65 / 15 months 7.38 / 15 months
El Matri L, et al.^41^	IVB + SML versus IVB 3 + PRN Jan 2015–Jan 2019	BCVA≦20/25 Presence of IRF and/or SRF	Retrospective 12 months	n = 98 eyes (49 vs. 49) (63 patients) Treatment naïve for DME CRT≦500 µm, BCVA≧20/400	IVB 359.9 ⇒ 305.9 54.0 IVB + SML 479.1 ⇒ 289.6 189.5	0.60 ⇒ 0.49 0.11 0.69 ⇒ 0.50 0.19	7.2 / 12 months 4.1 / 12 months
Khattab AM et al. 42	IVA + SML versus IVA 3 + PRN Feb 2017–Dec 2018	CRT > 250 µm	Prospective 18 months	n = 54 eyes (27 vs. 27) (51 patients) Excluded Intravitreal injections within the preceding 6 months, CRT > 250 µm, BCVA: 20/400 – 20/40	IVA 462.0 ⇒ 249.5 212.5 IVA + SML 457.1 ⇒ 244.6 212.5	† 31.7 ⇒ 50.6 18.9 (0.378) † 35.0 ⇒ 54.8 19.8 (0.396)	7.3 / 18 months 4.1 / 18 months
Kanar HS et al. 44	IVA + SML versus IVA 3 + PRN Apr 2015–Nov 2017	20% increase in CRT 1 line decrease at BCVA	Prospective 12 months	n = 56 (28 vs. 28) Treatment naïve for DME CRT≧ 300 µm, BCVA:0.2–0.9	IVA 451.28 ⇒ 328.8 122.5 IVA + SML 466.07 ⇒ 312.0 154.1	0.38 ⇒ 0.20 0.18 0.40 ⇒ 0.17 0.23	5.39 / 2 months 3.21 / 12 months
Abouhussein MA et al. 43	IVA + SML vs IVA 3 + PRN period: not stated	CRT≧300 µm	Prospective 15 months	n = 40 (20 vs. 20) Treatment naïve for DME CRT≧300 µm, BCVA > 3/60	IVA 457.9 ⇒ 290.5 167.4 IVA + SML 469.6 ⇒ 288.5 181.1	0.70 ⇒ 0.24 0.46 0.76 ⇒ 0.20 0.56	8.4 / 15 months 7.5 / 15 months
This study	IVA + SL versus IVA 3 + PRN Sep 2016–Sep 2020	100 µm increase in CRT 2 line decrease at BCVA	Prospective 24 months	n = 51 (25 vs. 26) Excluded Intravitreal injections within the preceding 90 days, CRT > 300 µm, BCVA: 0.05–0.7	IVA 442.8 ⇒ 319.5 123.3 IVA + SL 472.8 ⇒ 329.5 143.3	0.37 ⇒ 0.32 0.05 0.48 ⇒ 0.28 0.20	5.86 / 24 months 6.05 / 24 months

*CRT* central retinal thickness; *BCVA* best corrected visual acuity; *LogMAR* logarithm of the minimum angle of resolution; *anti-VEGF* anti-vascular endothelial growth factor; *SML* subthreshold micropulse laser; *IVR* intravitreal injection of ranibizumab; *IVB* intravitreal injection of bevacizumab; *IVA* intravitreal injection of aflibercept; *3 + PRN* initial 3 monthly injections and pro re nata; *IRF* intraretinal fluid; *SRF* subretinal fluid; *DME* diabetic macular edema; *ETDRS* Early Treatment Diabetic Retinopathy Study.

*: This value is the difference between the mean baseline value and the mean final value, not the mean change from baseline to final.

^†^: BCVA are described in ETDRS letters in this column. Values of change of BCVA in parentheses are equivalent to BCVA (logMAR).

Although the results of the three prospective trials and our trial were comparable in treating DME with the 3 + PRN regimen of IVA, there were differences in the patients, methods, and the retreatment criteria. The number of anti-VEGF injections in our study was close to the real-world practice, and the retreatment criteria was also closer to those of the real world compared to three prospective trials. It is possible that the treatment in this study was undertreatment, and it is believed that sufficient injections of anti-VEGF agents were not performed. This may have led to the finding of no significant difference in the number of anti-VEGF injections.

In two of the three prospective trials, treatment naïve cases of DME were the subjects. Our study included cases with pretreatments although the effects had probably been eliminated at the beginning of our study. In all of the previous prospective trials, micropulse lasers were used as the SL. In our study, PASCAL with the EPM algorithm (n = 17) or micropulse laser (n = 6) was used for the SL treatment. We suggest that the differences in the patients and methods were the cause of the differences in the results between our study and the three previous prospective studies.

The fewer number of injections of anti-VEGF agents in our study was probably due to differences in the methods, and the fewer number was the cause of the slight changes of BCVAs and CRTs. In addition, the fact that the patients were not limited to treatment naïve cases is probably the cause of the lesser degree of changes of the BCVAs and the CRTs.

There is a certain amount of time needed to eliminate the effects of the prior treatments, but the effects of these treatments cannot be completely ruled out. There were 14 treatment naïve cases in the IVA monotherapy group and 9 treatment naïve cases in the IVA + SL combination therapy group. It should be noted that the number of cases was small and the reliability was not high, but there was no significant difference in the number of injections between the two groups at 48 weeks (4.25 vs. 4.33, *P* = 0.88) and 96 weeks (5.79 vs. 5.44, *P* = 0.74) even in the treatment naïve cases.

The way SL was used was different from that of three earlier prospective studies. In our study, PASCAL with EPM algorithm and micropulse laser were used for the SL because scars of both lasers were not visible on any ophthalmic examinations and they were considered to have equivalent SL effects. However, the exposure time of the micropulse laser was 0.2 ms (200 ms with 10% duty cycle), while that of the EPM was 20 ms even though it is a short pulse. Therefore, the effects of both may be different which may be a limitation in this study. A more recent prospective study compared the effects of these two types of lasers with a monotherapy protocol^46^. The authors concluded that the micropulse system improved the functional outcomes more than the PASCAL system^40^. However, even in these earlier studies, there was no significant difference in the effects. PASCAL with EPM in reducing the CRT more than micropulse laser 3 months after the SL treatment^40^.

In our study, PASCAL (n = 17) was used more than the micropulse laser (n = 6). There was no significant difference in the number of injections between these two methods but the number of cases using the micropulse laser was small and reliable evaluations were not possible.

Taken together, the differences between the PASCAL with EPM and micropulse laser do not appear to have a large effect on the number of injections.

This study was not limited to treatment-naïve cases, and the number of injections of anti-VEGF agents was not high and was close to the real-world medical treatments in Japan. Under such conditions, the combination of IVA injections with SL was not able to completely suppress the recurrence of ME after three consecutive loading IVA injections. In addition, the combined therapy did not lead to fewer injections at 48 and 96 weeks, and there was no significant difference between the BCVAs and CRTs in both groups. However, there have been numerous reports on the effectiveness of SL for DME^17–27^, and our findings do not reject the effectiveness of SL for DME. Although the range of indications for SL treatment may be smaller than those for anti-VEGF treatment, it may be necessary to clarify the conditions of the DME in determining which each type of treatment is more effective.

If retinal microaneurysms (MAs) are found 500 µm away from the fovea, direct photocoagulations of the MAs are performed first in our hospital protocol. In this study, photocoagulation of the retinal MAs was performed in 5 of 24 patients in the IVA monotherapy group and 8 of 25 patients in the combination therapy group prior to their enrollment (Supplementary Table S1). In this protocol, cases in which photocoagulation of the MAs was performed were excluded within 90 days before enrollment, but they were not excluded no matter how many times they had been photocoagulated (Supplementary Table S3). If the ophthalmoscopic fundus examinations or fluorescein angiography reveals new MAs which increase the macular edema during the follow-up periods, photocoagulation of the MAs had been approved for use as a combination therapy. Photocoagulation of the MAs was performed in 2 of 24 patients in the IVA monotherapy group and 4 of 25 patients in the combination therapy group. It is possible that such treatment was the cause of the fewer number of anti-VEGF injections in this study. However, there was no significant difference in the number of cases treated with photocoagulations of MAs between the two groups prior to enrollment (*P* = 0.52) and during the follow-up periods (*P* = 0.67).

There are some limitations in this study. First, the number of cases analyzed was 49 (the cases of enrollment was 51) in both groups, which is small. Second, the total number of IVA monotherapy was fewer than that of previous similar studies. It is possible that this resulted in undertreatment of the DME, and there was no significant difference in the number of injections between the two groups. Third, two kinds of SL, PASCAL with the EPM algorithm and micropulse laser, were used. Both of these SL are effective on the DME but their mechanisms and the effects on the DME may be different. Thus, the conclusions of this study should be interpreted with caution.

In conclusion, the combined use of SL with IVA is as safe and effective for DME as IVA monotherapy. However, a synergistic effect was not found. In addition, the number of IVA cannot be reduced by the combination therapy.

## Patients and methods

### Ethics statements

The procedures used in this study were approved by the Institutional Review Board of the Chiba University Hospital, Japan (approval No. G27015, date of approval 22/07/2015) and the Chiba Rosai Hospital, Japan (approval No. 28-10, date of approval 06/10/2016). This study was registered in the University Hospital Medical Information Network Center (UMIN000019635, date of registration 09/11/2015). After establishing the Clinical Trials Act in Japan, this study was re-approved by the Chiba University certified Clinical Research Review Board (Approval No. CRB0002-18, date of approval 08/08/2018) and was registered in the Japan Registry of Clinical Trials (jRCTs031180182, date of registration 05/03/2019). Patients received a detailed explanation about the study and the procedures to be used, and all signed an informed consent form prior to their participation in the study. This study was conducted in compliance with the 1964 Declaration of Helsinki and its later amendments, “Ethical Guidelines on Medical Research for Humans” (Japan Ministry of Health, Labour and Welfare), and in accordance with the International Conference on Harmonization E6 Guideline for Good Clinical Practice.

### Study type and design

This was a Phase 4, randomized, open-label, active controlled, two-arm, multicenter, exploratory, investigator-driven clinical trial. This study was conducted in the Department of Ophthalmology, Chiba University and the Chiba Rosai Hospital, Chiba, Japan. Recruitment was started in September 2016, and the date on which the first patient was enrolled was 12 October 2016. The date of the last visit of the last patient was 23 September 2020.

### Patients

**Figure 5:**
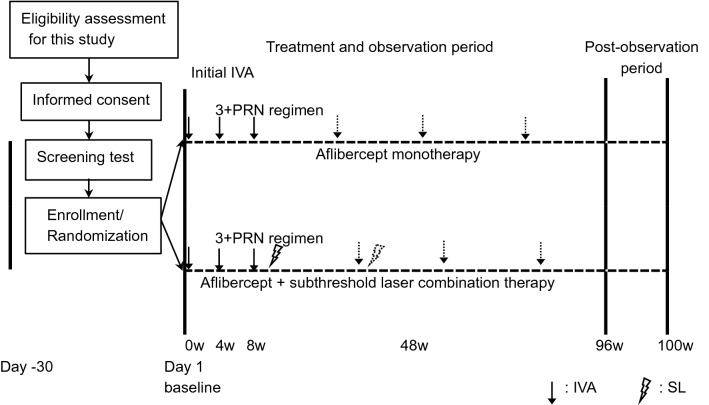
Flow diagram of study recruitment, randomization, study treatment and observations. *IVA* intravitreal aflibercept; *3* + *PRN*, initial 3 monthly injections and pro re nata; SL, subthreshold laser.

Patients were screened in either hospitals, and those meeting the inclusion criteria were enrolled. The follow-up period was 96 weeks after the first injection of the anti-VEGF agents (Day 1). The study design and flow diagram of the participants, randomization, study treatment, and observations are shown in Fig. 5.

The study populations consisted of Japanese male and female subjects who were older than 18 years and had type-1 or type-2 diabetes mellitus. Initially, 50 patients were enrolled. Both eyes were assessed at the screening examination for eligibility, and only one eye from each subject was randomly selected as the study eye. In cases where both eyes met the eligibility criteria, the study eye was the one with the greater macular thickness. The inclusion and exclusion criteria were assessed during the screening period which was within 30 days from Day 1 (Supplementary Tables S2 and S3). The decimal BCVAs was measured with a Landolt C chart at the screening examination, and they varied from 0.05 to 0.7 (20/400 to 20/29; 0.15 and 1.3 logMAR units). All of the CRTs at the screening period were > 300 µm. The sample size required for this study was determined based on the calculations from the results of the treatments performed at the Chiba University Hospital and considerations of the feasibility of the study. From the results using the ranibizumab different from this study, the 6-month success rate (no retreatment required) of the monotherapy group was estimated to be about 20%. When the 6-month success rate of the combination therapy group was estimated to be 50% and the sample size was 50, a 30% difference between the two groups can be detected during the observation period of 24 months. Because this was an exploratory test, the required sample size when using the log-rank test as the analysis of the primary endpoint with the two-tailed test, α = 0.1, 1 − β = 0.8 was 42 cases. From the existing studies, we assumed that the dropout rate was about 20%, and set the sample size in this study to 50.

After obtaining informed consent, the eligible patients were randomly assigned to the combination therapy group or the monotherapy group in an approximately 1:1 ratio by the pre-registered investigator. Case registration was performed based on a central registration system at the Clinical Research Data Center of the Clinical Research Center, Chiba University Hospital. The randomization sequence was generated using a computer-based dynamic allocation method with a minimization procedure to balance three allocation factors, viz., the age, baseline BCVA, and prior pan-retinal photocoagulation.

### Study of efficacy and safety of treatments

The primary outcome was the interval from the end of the initial 3 consecutive monthly loading IVA injections to the time before a re-injection was necessary. The secondary outcomes for efficacy were the number of IVA injections during the study period of 48 and 96 weeks, the mean changes in the central retinal thickness (CRT) from the baseline to that at 48 and 96 weeks, and the mean changes of the BCVA from the baseline to that at 48 and 96 weeks.

The secondary outcomes for safety were serious and minor adverse ocular and non-ocular events. For the evaluation of the adverse events, the latest version of the Medical Dictionary for Regulatory Activities presented by the International Council for Harmonization of Technical Requirements for Pharmaceuticals for Human Use was used.

The systemic blood pressures and pulse rates were determined before and after the IVA injections. The IOP was also measured before and after the IVA injections. Another adverse event which was examined was whether infectious diseases such as endophthalmitis developed.

### Treatments

All patients underwent central randomized placement (1:1 ratio) into two groups: Group 1, IVA monotherapy group received 2 mg/0.05 ml of intravitreal aflibercept (Eylea; Regeneron, Tarrytown, NY, and 75 Bayer Consumer Care AG, Basel, Switzerland), and Group 2 received the same dose of IVA and also had SL therapy.

The eligible patients in both groups receive monthly IVA injections for 3 months. The patients in Group 2 received SL within 4 weeks of the third monthly IVA injections. For the SL treatment, the PASCAL with the EPM algorithm was used at the Chiba University Hospital, and subthreshold micropulse laser was used at the Chiba Rosai Hospital. Randomization was balanced by the baseline decimal BCVA (< 0.4 or ≥ 0.4), age (< 63 or ≥ 63 years), and prior panretinal photocoagulation (+ or −).

After that, all patients were re-administered the PRN regimen (3 + PRN) according to the following retreatment criteria. All patients visited one of the two hospitals every 4 weeks and had a complete ophthalmologic examination including measurements of the BCVA, IOP, CRT, and slit-lamp examinations and dilated fundus examinations. The status of the DME was evaluated in the images obtained by spectral domain OCT (Heidelberg Engineering, Heidelberg, Germany/Carl Zeiss Meditech Inc., Dublin, California, USA). The CRT measurements were made with an automated measurements instrument used for the retinal map analysis protocol.

The efficacy of the treatments was based on the BCVAs and the CRTs.

### Laser treatment

For the SL treatment, the PASCAL with the EPM algorithm was used with the PASCAL streamline yellow (Topcon, Tokyo, Japan) at the Chiba University Hospital, and a subthreshold micropulse laser that delivered with IQ 577 (IRIDEX Corporation, Mountain View, California, USA) was used at the Chiba Rosai Hospital.

For the PASCAL with the EPM algorithm, a continuous wave 577 nm laser with short pulses was used. Initially, a test burn was made on an area outside the vascular arcade, and a subthreshold power was determined by titrating the burn to be barely visible. Then, the patients were treated with pulse grid pattern laser around the macula with the EPM algorithm which was 40% of the test burns. Four rows of laser were made around the macula using the Area Centralis contact lens (Volk Optical, Mentor, OH, USA). The treatment parameters were an exposure of 20 ms, spot diameter of 200 µm, and a spacing of 0.25 × spot diameter.

A 577-nm micropulse laser was used for the subthreshold micropulse laser burns. The minimal laser power that caused a just noticeable burn in the vascular arcade area using a 200 µm spot diameter and a 100 ms duration pulses in the continuous wave mode was determined. Then, the laser shots were applied with a 10% duty cycle micropulse mode at 200% of the threshold energy (120 to 170 mW) for 200 ms. This resulted in a delivery of 40% of the threshold energy^26,30^.

If an ME recurred in the combination therapy group, IVA was performed and then SL was performed within 4 weeks. However, SL can be performed only if there was an interval of ≥ 16 weeks from the previous SL, and it can be performed up to 2 times during the study period.

### Retreatment criteria

Retreatment was begun If any of the following criteria were met: CRT increased by ≥ 100 μm from the previous minimum value; the BCVA worsened by ≥ 0.2 logMAR units from the previous high value along with an increase in the CRT; and a new or persistent cystoid ME or subretinal fluid or persistent diffuse ME in the OCT images.

### Statistical analyses

The FAS included all randomized patients who received at least one intravitreal aflibercept except two patients who withdrew their consent prior to the start of the treatments. The PPS included all randomized patients excluding 2 cases from the FAS group that were discontinued before completion of induction treatment (three consecutive intravitreal aflibercepts in the monotherapy group, and subthreshold laser in addition to these in the combination therapy group). The FAS was used as data for statistical analyses for the primary outcomes. In principle, the PPS was used for the statistical analyses for secondary outcomes, but in the evaluation of the number of IVA during the periods of 48 and 96 weeks after the start of the study, cases in which the study was not completed were excluded. For the secondary outcomes for safety, the FAS was used.

The data are presented as the means ± SDs for the continuous variables and number and percentages for the categorical variables. Statistical comparisons of demographic data between groups were done using Student’s *t* tests, Chi-square tests, and Fisher exact tests. The primary outcome was analyzed using the Kaplan–Meier method and compared between groups using the log-rank test. For the statistical analyses, the decimal BCVA measured with a Landolt Chart was converted to logMAR units. Statistical comparisons of the secondary outcomes for efficacy were analyzed using the paired *t* tests, Poisson regression, and the linear mixed-effects model. Patients with no recurrence of ME for more than 16 weeks after the last IVA were allowed an 8-week visit interval. Therefore, the last value was carried over to the missing value. The method of LOCF was used for CRTs and BCVAs in the secondary outcomes. All statistical tests were 2-tailed, and a *P* value < 0.05 was considered statistically significant All analyses were performed using SAS version 9.4 (SAS Institute Inc., Cary, NC, USA).

### Additional analyses

To evaluate the validity of this study method, the monotherapy group and the combination group were divided into a group with a history of treatment for DME and a treatment naive group. To eliminate the effect of pretreatment on the DME, the number of injections in the treatment naïve monotherapy group (n = 14) and the treatment naïve combination group (n = 9) was compared.

## Supplementary Information

Supplementary Information.
