# Supplementary material for: Randomized clinical trial comparing intravitreal aflibercept combined with subthreshold laser to intravitreal aflibercept monotherapy for diabetic macular edema
Source: Sci Rep. 2022 Jun 23;12:10672. doi: 10.1038/s41598-022-14444-y (PMC9226173; doi:10.1038/s41598-022-14444-y)
Supplement: Supplementary file 1 — Supplementary Information. [file 41598_2022_14444_MOESM1_ESM.pdf]

Randomized Phase-4 Clinical Trial comparing intravitreal aflibercept combined with subthreshold laser photocoagulation to intravitreal aflibercept monotherapy for diabetic macular edema

Tomoaki Tatsumi[1,*], Yoko Takatsuna[1,2,*], Toshiyuki Oshitari[1,3], Tomomi Kaiho[1], Yohei Kawasaki[4,5], Yuki Shiko[4], Takeshi Sugawara[1,6], Takayuki Baba[1], Shuichi Yamamoto[1]

[1]Department of Ophthalmology and Visual Science, Chiba University Graduate School of Medicine, 1-8-1, Inohana, Chuo-ku, Chiba, Chiba, 260-8670, Japan

[2]Department of Ophthalmology, Chiba Rosai Hospital, 2-16, Tatsumidaihigashi, Ichihara, Chiba, 290-0003, Japan

[3]Department of Ophthalmology, International University of Health and Welfare School of Medicine, 4-3, Kozunomori, Narita, Chiba, 286-8686, Japan

[4]Biostatistics Section, Clinical Research Center, Chiba University Hospital, 1–8–1 Inohana, Chuo–ku, Chiba, 260–8670, Japan

[5]Faculty of Nursing, Japanese Red Cross College of Nursing, 4-1-3, Hiroo, Shibuya-ku, Tokyo, 150-0012, Japan.

[6]Chiba University Hospital Translational Research and Development Center, 1-8-1, Inohana, Chuo-ku, Chiba, Chiba, 260-8670, Japan

		IVA Monotherapy group (n=24)		IVA + SL Combination therapy group (n=25)		***P*-value**
Sex	Male, n (%)	16	(66.7)	11	(44.0)	Fisher
	Female, n (%)	8	(33.3)	14	(56.0)	*P*=0.154
Age, years	Mean (S.D)	69.3	(7.4)	66.9	(9.4)	*t* test (equal) *P*=0.323
	＜50 , n (%)	0	(0)	2	(8.0)	Chi-square test
	50≦ ＜60, n (%)	3	(12.5)	2	(8.0)	*P*=0.487
	60≦ ＜70, n (%)	10	(41.7)	12	(48.0)	
	70≦ ＜80, n (%)	9	(37.5)	7	(28.0)	
	80≦ , n (%)	2	(8.3)	2	(8.0)	
Subject eye	Right, n (%)	14	(58.3)	10	(40.0)	Fisher
	Left, n (%)	10	(41.7)	15	(60.0)	*P*=0.258
Mean interval between DME diagnosis and first IVA, months	Mean (S.D)	19.8	(36.8)	23.9	(33.2)	*t* test (equal) *P*=0.685
	＜1 , n (%)	4	(16.7)	0	(0)	Chi-square test
	1≦ ＜7 , n (%)	12	(50.0)	8	(32.0)	*P*=0.029
	7≦ ＜27 , n (%)	3	(12.5)	10	(40.0)	
	27≦ , n (%)	5	(20.8)	7	(28.0)	
HbA1c, %	Mean (S.D)	7.20	(0.79)	7.41	(1.41)	*t* test (unequal) *P*=0.485
	＜6.5	5	(20.8)	6	(24.0)	Chi-square test
	6.5 ≦ ＜7.2	6	(25.0)	8	(32.0)	*P*=0.934
	7.2 ≦ ＜7.7	5	(20.8)	4	(16.0)	
	7.7 ≦	8	(33.3)	7	(28.0)	
Hypertension	(+), n (%)	11	(45.8)	10	(40.0)	Fisher
	(-), n (%)	13	(54.2)	15	(60.0)	*P*=0.776
eGFR, mL/min/1.73m^2	Mean (S.D)	62.5	(20.6)	72.9	(20.5)	*t* test (unequal)
						P=0.090
	＜30, n (%)	1	(4.2)	0	(0)	Chi-square test
	30≦ ＜50, n (%)	5	(20.8)	3	(12.0)	*P*=0.391
	50≦ , n (%)	18	(75.0)	22	(88.0)	
Prior photocoagulation, n (%)	PRP	9	(37.5)	10	(40.0)	Chi-square test
	Focal PC	1	(4.2)	4	(16.0)	*P*=0.334
	none	14	(58.3)	11	(44.0)	
Prior DME, n (%)	treat	11	(45.8)	18	(72.0)	Chi-square test *P*=0.085
	treat: Anti-VEGF	2		4		
	treat: Local TA	8		11		
	treat: MAPC	5		8		
	not treat	13	(54.2)	7	(28.0)	
Lens status, n (%)	phakia	20	(83.3)	16	(65.2)	Fisher
	pseudophakia	4	(16.7)	9	(34.8)	*P*=0.196
CRT, μm	Mean (S.D)	442.8	(91.3)	476.4	(136.1)	*t* test (equal) *P*=0.377
	＜ 389	6	(25.0)	6	(24.0)	Chi-square test
	389 ≦ ＜433	5	(20.8)	8	(32.0)	*P*=0.303
	433 ≦ ＜509	8	(33.3)	3	(12.0)	
	509 ≦	5	(20.8)	8	(32.0)	
BCVA (logMAR)	Mean (S.D)	0.369	(0.235)	0.478	(0.320)	*t* test (equal) *P*=0.188
	＜ 0.221	4	(16.7)	3	(12.0)	Chi-square test
	0.221 ≦ ＜0.301	5	(20.8)	3	(12.0)	*P*=0.762
	0.301 ≦ ＜0.522	8	(33.3)	11	(44.0)	
	0.522 ≦	7	(29.2)	8	(32.0)	
IOP, mmHg	Mean (S.D)	13.5	(3.0)	13.5	(3.0)	*t* test (equal) *P*=0.943
	＜11	4	(16.7)	4	(16.0)	Chi-square test
	11≦ ＜14	7	(29.2)	7	(28.0)	*P*=0.612

	14≦ <16	5	(20.8)	9	(36.0)	
	16≦	8	(33.3)	5	(20.0)	
Hard exudate in macula	(-), n (%)	21	(87.5)	20	(80.0)	Fisher
	(+), n (%)	3	(12.5)	5	(20.0)	*P*=0.702
Macular hemorrhage	(-), n (%)	19	(79.2)	23	(92.0)	Fisher
	(+), n (%)	5	(20.8)	2	(8.0)	*P*=0.265
Vitreous hemorrhage	(-), n (%)	23	(95.8)	25	(100.0)	Fisher
	(+), n (%)	1	(4.2)	0	(0)	*P*=0.490
Retinal neovascularization	(-), n (%)	22	(91.7)	23	(92.0)	Fisher
	(+), n (%)	2	(8.3)	2	(8.0)	*P*=0.100

Supplementary Table S1. The baseline characteristics of patients (FAS).
FAS, full analysis set; IVA, intravitreal injection of aflibercept; SL, subthreshold laser; SD, standard deviation; eGFR, estimated glomerular filtration rate; CRT, central retinal thickness; BCVA, best-corrected visual acuity; logMAR, logarithm of minimum angle of resolution; IOP, intraocular pressure; PRP, panretinal photocoagulation; PC, photocoagulation; VEGF, vascular endothelial growth factor; TA, triamcinolone acetonide injection; MAPC, microaneurysm photocoagulation.

1)	Japanese male and female ≥ 18 years with type 1 or 2 diabetes mellitus.
2)	DME with central involvement in the study eye
3)	Decrease in visual acuity determined to be primarily the result of DME in the study eye.
4)	Central macular thickness ≥ 300 μm in the study eye.
5)	BCVA at study entry from 0.7 to 0.05 (decimal) in the study eye.
6)	Patients who have received sufficient explanation to participate in the study and have given written consent with sufficient understanding and free will.
7)	When both eyes met the above criteria, the eye with the larger retinal thickness.

Supplementary Table S2. Inclusion criteria
DME, diabetic macular edema; OCT, optical coherence tomography; BCVA, best corrected visual acuity.

1)	Patients with a history of treatment with vitrectomy or buckling surgery in the study eye.
2)	Patients with a history of treatment with filtration surgery for glaucoma or possibility of glaucoma surgery in the future in the study eye.
3)	Patients with active proliferative diabetic retinopathy (PDR) in the study eye.
4)	Patients with medical history of idiopathic or autoimmune uveitis in the study eye.
5)	Patients who have vitreomacular traction syndrome or epiretinal membrane, which significantly affects visual acuity of the study eye.
6)	Patients with iris neovascularization, vitreous hemorrhage, traction retinal detachment in the study eye.
7)	Patients with preretinal fibrosis extending to macular in the study eye.
8)	Patients with structural damage of central macular preventing improvement of visual acuity after regression of macular edema, such as atrophy of retinal pigment epithelium, subretinal fibrosis or scar, significant macular ischemia, hard exudate.
9)	Patients with possibility of low vision to conduct medical or surgical interventions during the study period, complications which should affect the study result.
10)	Patients with a history of treatment with cataract surgery or other intraocular surgery within 90 days of day 1.
11)	Patients with a history of treatment with laser photocoagulation (pan-retinal or macular) in the study eye within 90 days of day 1.
12)	Patients with a history of treatment with posterior capsulotomy with yttrium aluminum garnet (YAG) laser treatment within 30 days of day 1.
13)	Patients with previous use of intraocular or periocular corticosteroids in the study eye within 120 days of day 1.
14)	Patients who have received macular laser treatment (excluding direct coagulation for capillary aneurysms) three or more times in the past, or patients who are judged to be unable to expect the effects of laser treatment.
15)	Patients with a history of treatment with anti-VEGF agents in the study eye (pegaptanib sodium, bevacizumab, ranibizumab) within 90 days of day 1.
16)	Patients with symptoms of infectious blepharitis, keratitis, scleritis or conjunctivitis in either eye.
17)	Patients with insufficient degree of clearness of optic media for the fundus examination or OCT imaging.
18)	Patients currently being treated for serious systemic infections.
19)	Patients with uncontrolled diabetes mellitus (HbA1c > 12.0%).
20)	Patient with poor controlled hypertension (sitting systolic pressure >160 mmHg or diastolic pressure >95 mmHg).
21)	Patients with renal failure indicating dialysis or renal transplantation.
22)	Patients with events of cerebrovascular disease or myocardial infarction within 180 days of day 1.
23)	Patients with systemic treatments of anti-VEGF agents within 180 days of day 1.
24)	High risk patients, who will affect study results or develop complications, based on medical records, metabolic dysfunction, or laboratory test finding s indicating a disease and/or conditions for that the test drug will be contraindicated.
25)	Patients with allergy to fluorescein.
26)	Female patients who are pregnant or breastfeeding or want to become pregnant during the study period.
27)	Patients judged not to be adequate by the investigator.

Supplementary Table S3. Exclusion criteria
OCT, optical coherence tomography; VEGF, vascular endothelial growth factor.

a

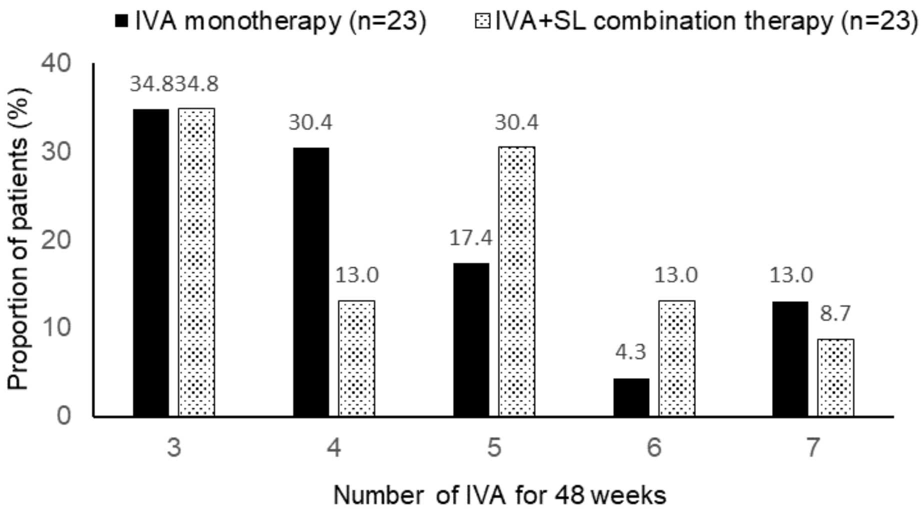

b

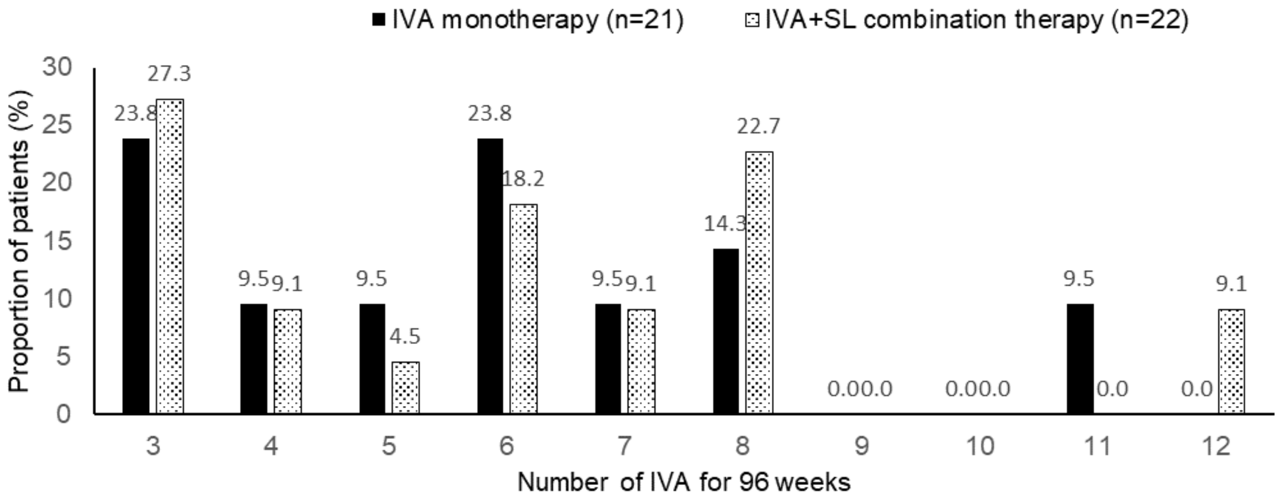

Supplementary Figure S1

(a) Proportion of patients per number of IVA for 48 weeks, (b) Proportion of patients per number of IVA for 96 weeks.
